# Role of TRPV1 in the Differentiation of Mouse Embryonic Stem Cells into Cardiomyocytes

**DOI:** 10.1371/journal.pone.0133211

**Published:** 2015-07-24

**Authors:** Yan Qi, Zenghua Qi, Zhichao Li, Chun-Kit Wong, Chun So, Iek-Chi Lo, Yu Huang, Xiaoqiang Yao, Suk-Ying Tsang

**Affiliations:** 1 Li Ka Shing Institute of Health Sciences and School of Biomedical Sciences, Chinese University of Hong Kong, Hong Kong, China; 2 School of Life Sciences, Chinese University of Hong Kong, Hong Kong, China; 3 Partner State Key Laboratory of Agrobiotechnology, Chinese University of Hong Kong, Hong Kong, China; 4 Centre of Novel Biomaterials, Chinese University of Hong Kong, Hong Kong, China; University of Pecs Medical School, HUNGARY

## Abstract

Cytosolic Ca^2+^ ([Ca^2+^]_i_) is an important signal that regulates cardiomyocyte differentiation during cardiogenesis. TRPV1 is a Ca^2+^-permeable channel that is expressed in cardiomyocytes. In the present study, we utilized mouse embryonic stem cell-derived cardiomyocytes (mESC-CMs) as a model to investigate the functional role of TRPV1 in cardiomyocyte differentiation. Induction of embryonic stem cells into cardiomyocytes was achieved using embryoid body (EB)-based differentiation method. Quantitative PCRs showed an increased TRPV1 expression during the differentiation process. In [Ca^2+^]_i_ measurement study, application of TRPV1 agonists, capsaicin and camphor, elicited a [Ca^2+^]_i_ rise in mESC-CMs, the effect of which was abolished by TRPV1-shRNA. In functional study, treatment of EBs with TRPV1 antagonists (capsazepine and SB366791) and TRPV1-shRNA reduced the size of the EBs and decreased the percentage of spontaneously beating EBs. TRPV1 antagonists and TRPV1-shRNA also suppressed the expression of cardiomyocyte marker genes, including cardiac actin, c-TnT, c-TnI, and α-MHC. Taken together, this study demonstrated an important functional role of TRPV1 channels in the differentiation of mESCs into cardiomyocytes.

## Introduction

Embryonic stem cells (ESCs) are derived from the inner cell mass of blastocysts. They can self-renew and are pluripotent, meaning that they can proliferate indefinitely and have the ability to differentiate into different cell lineages including cardiomyocytes [1]. ESC-derived cardiomyocytes (ESC-CMs) not only provide an unlimited *ex vivo* source of cardiomyocytes for cell-based heart therapies, but also are an excellent model for studying heart development. Ultrastructural, molecular biological, and electrophysiological studies have demonstrated that *in vitro* differentiation of mESC-CMs within the EBs closely recapitulates the developmental pattern of murine embryonic cardiomyocytes [2–4], making mouse ESCs (mESCs) an attractive model for investigating early cardiomyogenesis [2–4].

[Ca^2+^]_i_ is an important second messenger that regulates the differentiation and proliferation of cardiomyocytes during cardiogenesis [5]. During ESC differentiation into cardiomyocytes, there is a drastic change in expressional profile of some key Ca^2+^-handling proteins. For example, in undifferentiated mESCs, only inositol trisphosphate receptors but not ryanodine receptors are expressed in endoplasmic reticulum [6,7], and the main Ca^2+^ influx pathway is store-operated Ca^2+^ entry but not voltage-operated Ca^2+^ entry [6,8,9]. Furthermore, during cardiomyocyte differentiation, the expressions of ryanodine receptors, voltage-dependent Ca^2+^ channels and sarcoplasmic reticulum Ca^2+^-pump are increased [2,5,10,11]. These changes in the expression profile of Ca^2+^-handling proteins could alter temporal and spatial pattern of Ca^2+^ signaling, thereafter regulates the cardiomyocyte differentiation. Indeed, it is reported that Ca^2+^ entry through T-type voltage-gated Ca^2+^ channels may promote ESC differentiation into cardiomyocytes [12,13], whereas CD38-cADPR-Ca^2+^ signaling pathway may antagonize ESC differentiation into cardiomyocytes [14].

Transient receptor potential channel vanilloid 1 (TRPV1/VR1) is a member of the TRP super family of ion channels. It is a Ca^2+^-permeable cationic channel with predicted topology of six transmembrane segments (S1–S6) and a pore region between S5 and S6 [15]. The channel is widely expressed in variety of different tissues and cell types including nervous system, vascular cells, adipocytes and keratinocytes [16]. Functionally, TRPV1 is involved in pain sensation, thermosensation, inflammation, vascular tone control and adipogenesis [16]. TRPV1 is expressed in cardiomyocytes. However, its function in cardiomyocytes is poorly understood [17,18]. Up to the present, there is only one report about TRPV1 function in cardiomyocytes, in which TRPV1 is suggested to be involved in cold temperature-induced cardiac remodeling [17]. In the present study, we utilized mESC-CMs as the model to explore the possible role of TRPV1 in mESC differentiation into cardiomyocytes. Our results suggest an important role of TRPV1 in mESC differentiation into cardiomyocytes.

## Materials and Methods

### Culture of mESCs

The mESC line D3 (ATCC, Manassas, VA) was used in this study. It was cultured on the 6-well plates coated with 0.1% of gelatin without feeder layer. The mESCs were maintained in Dulbecco’s modified Eagle’s medium (DMEM) (Invitrogen, Carlsbad, CA) supplemented with 15% fetal bovine serum (Hyclone, Thermo Fisher Scientific Inc., Waltham, MA), 2 mM L-glutamine (Gibco, Carlsbad, CA), 0.1 mM mercaptoethanol (Sigma, St. Louis, MO), 0.1 mM non-essential amino acids (Gibco), and 1,000 U/ml leukemia inhibitory factor (Chemicon, Millipore, Billerica, MA) [19].

### Differentiation of mESCs into cardiomyocytes

The mESCs were resuspended in normal differentiation medium which had the same components as the medium for undifferentiated mESCs but without leukemia inhibitory factor. EBs were formed through hanging drop method as described by us previously [20]. Briefly, mESCs were spotted in droplets onto the lid of 90-mm petri dishes and incubated upside down for 2 days to allow the formation of the EBs. Each droplet contained 800 cells in 20 μl of differentiation medium. The bottom of petri dish was covered by 10 ml of phosphate-buffered saline (PBS) to avoid drying of the hanging drops. On the differentiation day 2 (day 2), EBs were washed into 10-cm petri dishes with 10 ml differentiation medium and cultured in suspension for another 5 days. In the experiments examining the effect of TRPV1 antagonists on cardiomyocyte differentiation, TRPV1 antagonists or 0.1% DMSO (as vehicle control) were included from day 2 to day 7, which is the crucial period for cardiomyocyte differentiation.

On differentiation day 7, the sizes of EBs were measured using SPOT Advanced imaging software (Diagnostic Instruments, Inc., Sterling Heights, MI). The EBs were then attached onto a 24-well gelatin-coated culture plate containing differentiation medium for 10 more days (until day 17). Spontaneous beating of the EBs was monitored under microscope.

### Lentiviral TRPV1-shRNA

A short hairpin RNA (shRNA) construct against mouse TRPV1 was generated using pLKO.1 puro vector containing a cassette for puromycin according to the manufacturer’s instructions (OligoEngine, USA). The targeted sequences used were 5’-GCCAGACAGCATTACACATTG-3’. Scrambled shRNA was used as control. The oligonucleotide insertion was confirmed by sequencing. Lentiviral production was done by co-transfecting pLKO.1 and packaging vectors into HEK293FT cells. The supernatants were collected after 48 hr. The viruses were purified by centrifuging for 2 hr at 50,000 g and then resuspended in PBS.

Lenti-TRPV1-shRNA and its scrambled control were stably transduced into mESCs. After puromycin selection, two mESC lines were established, one stably expressing TRPV1-shRNA and the other expressing its scramble control. These mESC lines were then differentiated into cardiomyocytes.

### Immunostaining

Undifferentiated mESCs or differentiated cells were cultured on glass coverslips and fixed with 4% paraformaldehyde in PBS for 20 min and permeabilized with 0.1% Triton X-100 in PBS for 15 min before blocking with 2% BSA solution for 1 hr at room temperature. The samples were incubated with the primary antibodies: the goat anti-TRPV1 (1:50 dilution), or the mouse anti-Oct4 (1:200 dilution), or the mouse anti-cTnT (1:500 dilution) at 4°C overnight. The samples were then incubated with the secondary antibodies: AlexaFluor 488 donkey anti-mouse IgG and AlexaFluor 546 rabbit anti-goat IgG (1:1000 dilution). Nuclei were counterstained with DAPI (Invitrogen). In control experiments, the primary antibodies were either omitted or were preabsorbed for 2 hr at room temperature with excessive of TRPV1 peptides (at 1:1 weight ratios) provided by manufacturer. The cells were visualized under a confocal microscope (Olympus FV1000).

### Isolation of mESC-CMs and [Ca^2+^]_i_ measurement

EB dissection and cardiomyocyte isolation were performed 2 days before [Ca^2+^]_i_ measurement. The methods for EB dissection and cardiomyocyte isolation were described elsewhere [21]. Beating regions were isolated by using 1 ml syringe with 27-G needle under dissection microscope. Dissected regions were placed in sterile 1.5 ml eppendorf with differentiation medium. The dissected regions were centrifuged at 1000 rpm, 4°C for 5 min. Then, the medium was removed and the cell pellet was washed with PBS. The pellet was centrifuged at 1000rpm, 4°C for 5 min and PBS was removed. The cell pellet was digested for 30 min at 37°C in an air shaker (150 rpm) with a digestion solution that contains: 1 mg/ml collagenase B (Roche Diagnostics, USA), 120 mM NaCl, 5.4 mM KCl, 5 mM MgSO_4_, 5 mM Na Pyruvate, 20 mM glucose, 20 mM taurine, 10 mM HEPES and 30 μM CaCl_2_, pH adjusted to 6.9 by NaOH. Afterward, the cells were centrifuged down and recovered for 1 hr in KB solution that contains 85 mM KCl, 30 mM K_2_HPO_4_, 5 mM MgSO_4_, 1 mM EGTA, 5 mM pyruvic acid, 5 mM creatine, 20 mM taurine, 20 mM glucose, 87 μM of Na-ATP, pH adjusted to 7.2 by NaOH. After recovery, spontaneously beating cells in KB solution were seeded on glass slides pre-coated with 20 μg/ml laminin and 0.1% gelatin.

Cells were loaded with 5 μM Fluo-4 and 0.02% pluronic acid F-127 in Tyrode’s solution in dark for 20 min at 37°C. The dye was washed away and the cells were incubated in Tyrode’s solution. Tyrode’s solution contained: 140 mM NaCl, 1.8 mM CaCl_2_, 1 mM MgCl_2_, 5.4 mM KCl, 10 mM glucose, 10 mM Hepes, pH adjusted to 7.2 by NaOH. Ca^2+^-free Tyrode’s solution contained additional 3 mM EGTA. Fluorescence images were collected every 17 to 18 msec using an Olympus FluoView FV1000 laser scanning system (Olympus, Tokyo, Japan) equipped with an argon laser of 488 nm. Ca^2+^ change were expressed as ratio of fluorescence relative to the intensity immediately before capsaicin or camphor challenge (F1/F0).

### Immunostaining followed by flow cytometry

Flow cytometer was used to analyze c-TnT-positive cardiomyocytes. Briefly, EBs were digested into single cell suspension. The cells were washed with PBS and trypan blue exclusion assays were performed. 1 × 10^6^ viable cells were first fixed with 4% paraformaldehyde in PBS and permeabilized with 0.1% Triton X-100 in PBS. The cells were then blocked with 0.5% bovine serum albumin/5% normal goat serum in PBS. Mouse anti-cTnT antibody (Abcam) was then added to the cells at 1:200 dilution in blocking buffer for 1 hr at room temperature. After washing with PBS, the cells were incubated with Alexa Fluor 488-conjugated goat anti-mouse antibody (Life Technologies) at 1:100 dilution in 0.5% bovine serum albumin/1% normal goat serum in PBS for 45 min at room temperature. After the final wash with PBS, the cells were analyzed with FACSCanto flow cytometer (BD). Data from 10,000 single cell events were collected and analyzed with FACSDiva software (BD).

### Quantitative real-time polymerase chain reaction (qPCR)

Some EBs were collected on differentiation day 12 and day 17 for qPCR. Total RNA was extracted using Trizol reagent according to the manufacturer’s protocol. The extracted RNA was treated with DNase I and subjected to reverse transcription using SuperScript First-Strand Synthesis System. Power SYBR GREEN PCR Master Mix (Applied Biosystems, Foster City, CA) was used for qPCR. PCR primers were: TRPV1 forward CCGGCTTTTTGGGAAGGGT, reverse GAGACAGGTAGGTCCATCC; beta-actin forward AGAGGGAAATCGTGCGTGAC, reverse CAATAGTGATGACCTGGCCGT; cardiac actin forward CCAGCCCAGCTGAATCC, reverse CCATTGTCACACACCAAAGC; c-TnT forward TTCATGCCCAACTTGGTGCC, reverse CTCTCTTCAGCCAGGCGGTTC; cardiac troponin I (c-TnI) forward AGGGCCCACCTCAAGCA, reverse GGCCTTCCATGCCACTCA; alpha-myosin heavy chain (α-MHC) forward, AGCTGACAGGGGCCATCAT, reverse ACATACTCGTTCCCCACCTTC. The qPCR reactions were performed with 7500 Fast Real-time PCR system (Applied Biosystems). Each reaction was performed under the following conditions: 50°C for 2 min; 95°C for 10 min; 40 cycles of 95°C for 15 sec, 60°C for 1 min. Melting curve analysis of PCR products were performed to verify the authenticity of PCR products. All samples were run at least in triplicate. Fold changes in the relative mRNA expression of the target genes were determined using the 2^-ΔΔCt^ method [22], in which the target genes were normalized to the house-keeping gene and the relative expression levels of different genes in experimental groups were normalized to that in control group.

## Results

### Expression of TRPV1 channels in mESCs and mESC-CMs

**Fig 1:**
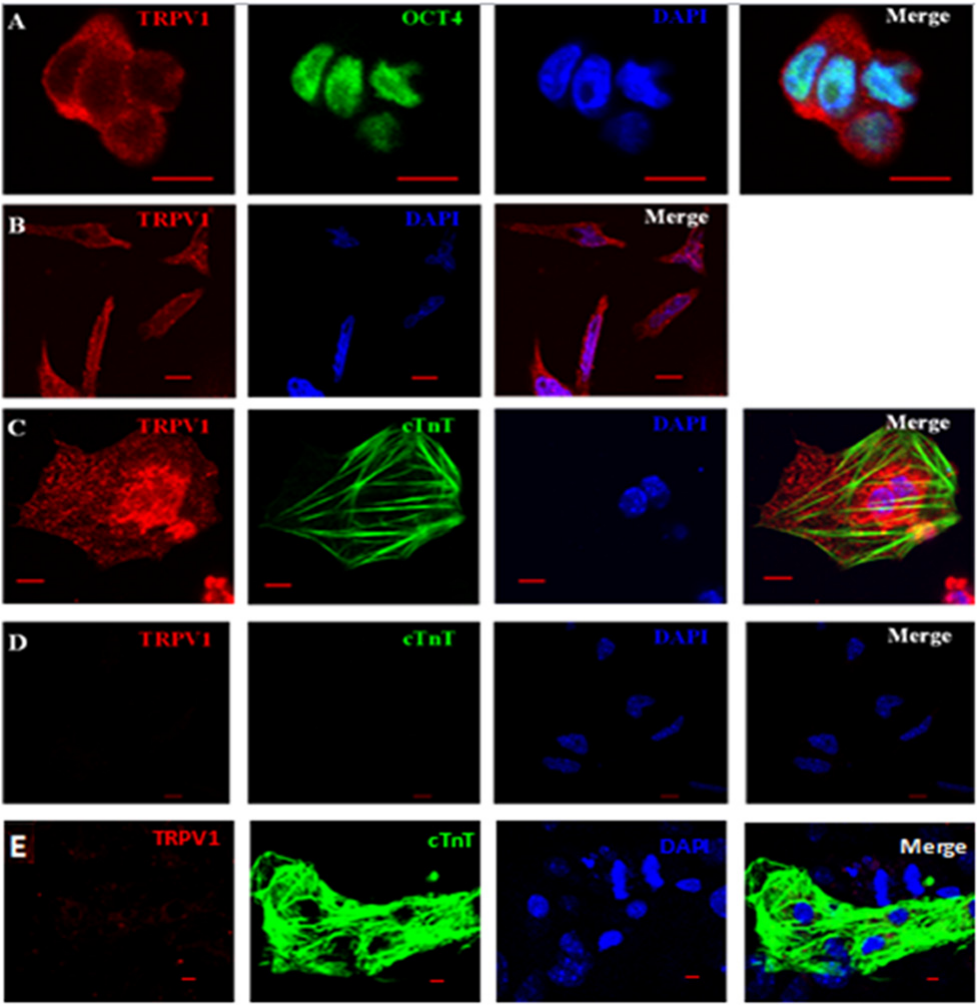
Expression of TRPV1 in mESCs and mESC-CMs. (A) Representative immunostaining images of undifferentiated mESCs (day 0). Oct4 (green) was a marker of undifferentiated MSCs. (B) Representative immunostaining images of differentiating mESCs (day 4). (C) Representative images from differentiated mESC-CMs (day 9). c-TnT was a cardiomyocyte marker. (D) Negative control in the absence of primary antibody for day 9 cells. (E) Negative control after TRPV1-peptide preabsorption. A through E, from left to right, were images of TRPV1 (red), Oct4/c-TnT (green), DAPI (blue) and overlay. Scale bars, 10μm. Experiments were performed 3–5 times.

Immunostaining was used to examine the expression of TRPV1 proteins in mESCs and mESC-CMs. The cells were stained on day 0 (undifferentiated mESCs), day 4 (differentiating mESCs) and day 9 (differentiated mESC-CMs). OCT4 (octamer-binding transcription factor 4) is a transcription factor that is required to maintain the self-renewal of ESCs. As expected, undifferentiated mESCs were shown to express OCT4 on day 0 (Fig 1A, green). On the differentiation day 9, ~10% of cells were stained positive for c-TnT, a cardiomyocyte-specific marker (Fig 1C, green). TRPV1 immunoreactivity (in red) was detected in cells of all differentiation stages from undifferentiated mESCs (day 0), to differentiating EBs (day 4), and differentiated mESC-CMs (day 9). Two sets of negative control experiments were done, one performed in the absence of the primary anti-TRPV1 antibodies (Fig 1D) and the other with antigen (TRPV1-peptide) preabsorption (Fig 1E). Very little TRPV1 immunoreactivity could be detected in either negative control (Fig 1D and 1E).

**Fig 2:**
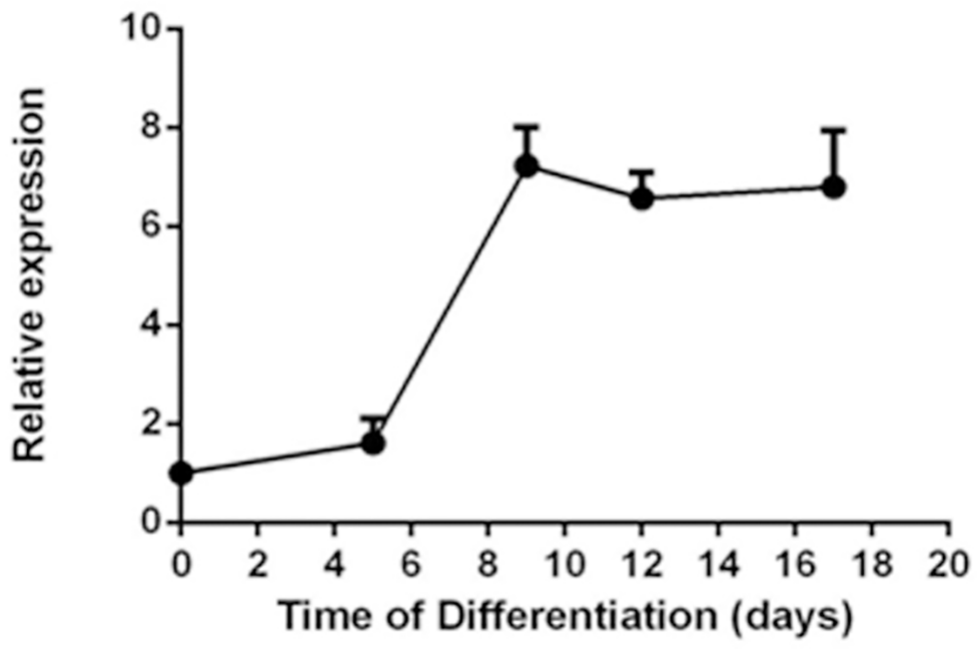
Expression pattern of TRPV1 mRNA during the differentiation of mESCs to cardiomyocytes. Shown was the relative expression of TRPV1 mRNA at different differentiation days. Values were Mean ± SEM. n = 3.

Quantitative PCRs were performed to determine the expression pattern of TRPV1 mRNA during the cardiomyocyte differentiation process. The results showed an increase in TRPV1 expression during the differentiation process (Fig 2).

**Fig 3:**
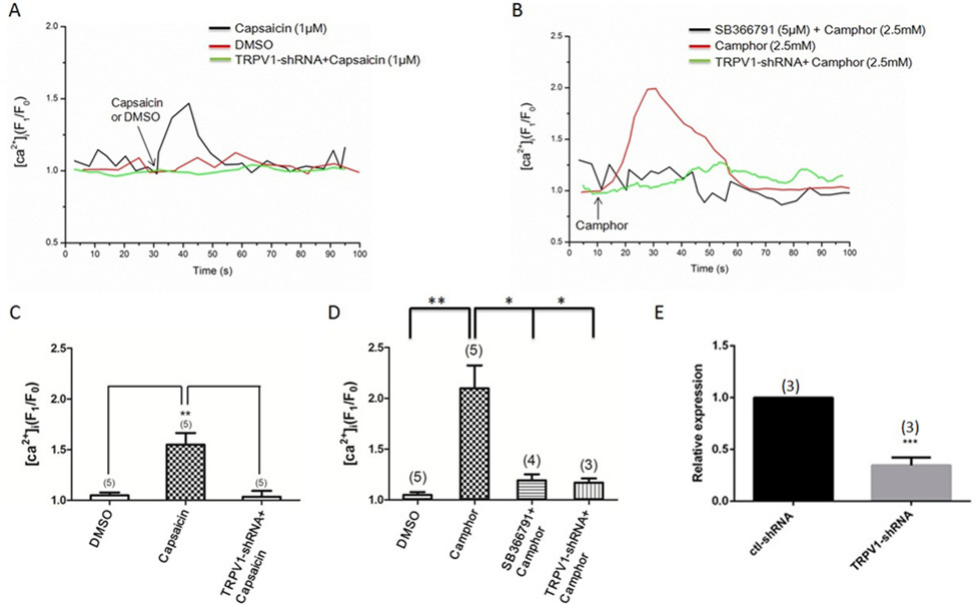
Capsaicin and camphor-induced rise in basal [Ca^2+^]i level in mESC-CMs. (A and B) Representative traces showing the [Ca^2+^]i responses to 1 μM capsaicin (A) and 2.5 mM camphor (B). (C and D) summary of data comparing the maximal [Ca^2+^]i, while rise in response to 1 μM capsaicin (C) and 2.5 mM camphor (D). Shown were effects of TRPV1-shRNA and SB366791 (10 μM). (E) Effectiveness of TRPV1-shRNA in suppressing TRPV1 mRNA by qPCR. ctl-shRNA stands for control scrambled shRNA. Values were Mean ± SEM. n = 3–5 experiments as labeled. * *P* < 0.05, ** *P* < 0.01, ***P < 0.001.

Functional presence of TRPV1 in mESC-CMs was verified by [Ca^2+^]_i_ measurement study. Application of a TRPV1 agonist capsaicin (1 μM) elicited a rise in basal [Ca^2+^]_i_, while in ~50% of individual mESC-CMs (Fig 3A and 3C). In other 50% of mESC-CMs, capsaicin failed to cause an [Ca^2+^]_i_, while increase. Camphor (2.5 mM), another TRPV1 agonist, caused a similar rise in basal [Ca^2+^]_i_, whil e in ~50% of individual mESC-CMs, the effect of which was inhibited by a selective TRPV1 antagonist SB366791 (10 μM) (Fig 3B and 3D). A TRPV1-shRNA was generated. Lentiviral mediated delivery of the TRPV1-shRNA reduced the TRPV1 expression in mESC-CMs by ~70% (Fig 3E). TRPV1-shRNA almost abolished the capsaicin- and camphor-induced [Ca^2+^]_i_, while rises in mESC-CMs (Fig 3C and 3D). In the absence of bath Ca^2+^, while, the magnitude of [Ca^2+^]_i_, while rise to camphor was much smaller (~8% rise in [Ca^2+^]_i_, while for the cells bathed in Ca^2+^-free Tyrode solution *vs*. ~100% rise in [Ca^2+^]_i_, while for the cells bathed in Ca^2+^-containing Tyrode solution, *n* = 3), suggesting that the [Ca^2+^]_i_, while rise was mostly due to extracellular Ca^2+^, while entry. Furthermore, capsaicin failed to induce cytosolic Ca^2+^ rise in undifferentiated mESCs. This agrees well the expression profile of TRPV1 during differentiation (Fig 2), which showed low TRPV1 expression in undifferentiated mESCs.

### Effect of TRPV1 antagonists the growth of EBs

**Fig 4:**
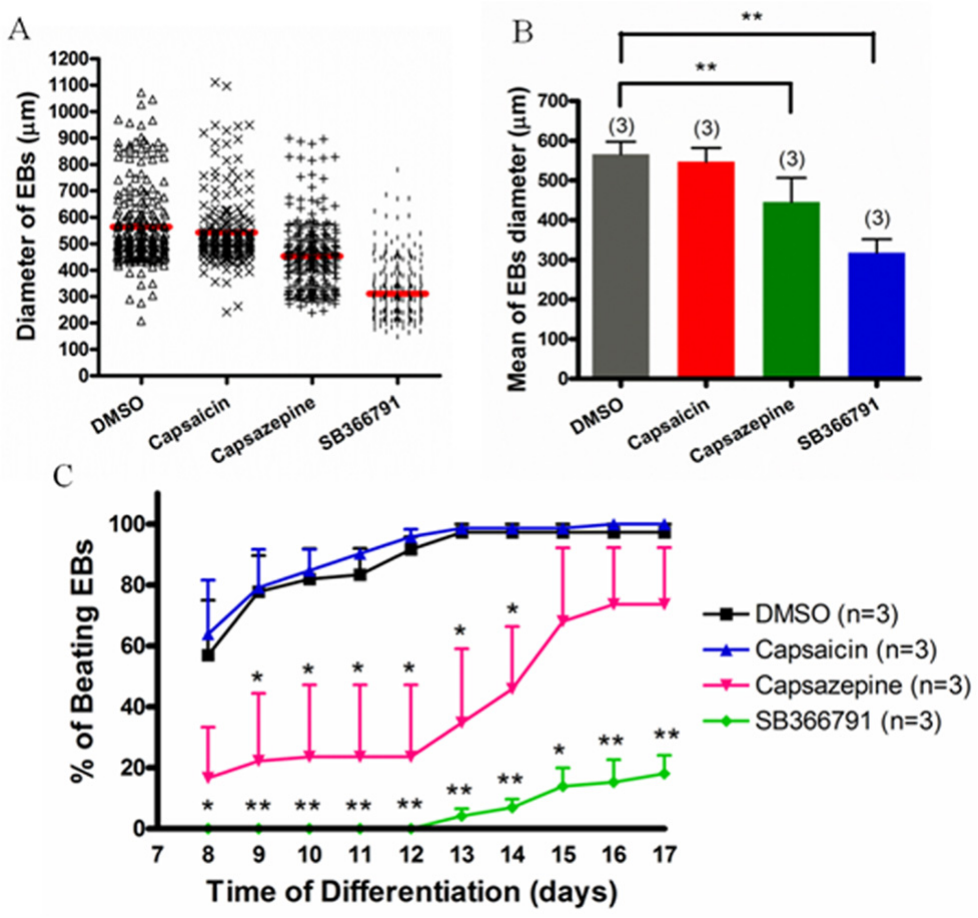
Effect of TRPV1 agonist or antagonists on EB sizes and EB beating curves. (A) (A) Summary of data illustrating EB size distribution on the differentiation day 7 after treatment with 0.1% DMSO (vehicle control), 1 μM capsaicin, 10 μM capsazepine, or 10 μM SB366791. Each group contained more than 200 EBs from 3 independent experiments, with red bar representing the average size. (B) Data summary of EB sizes under different treatments. (C) The percentage of beating EBs after different treatments. The beating was recorded from differentiation day 8 to day 17. Results were Mean ± SEM. n = 3 experiments. * *P* < 0.05, ** *P* < 0.01 compared to DMSO control.

The diameters of EBs were measured by SPOT Advanced imaging software on differentiation day 7. In vehicle control group treated with 0.1% DMSO, EBs appeared round-shaped with clear edge. The mean EB diameters were 566 ± 31 μm. Capsazepine (10 μM) and SB366791 (10 μM) treatments reduced the diameter of EBs to 446 ± 61 μm and 318 ± 34 μm, respectively (Fig 4A and 4B), while capsaicin (1 μM), a putative TRPV1 agonist, had no effect on the diameter (547 ± 35 μm) of the EBs (Fig 4A and 4B).

### Effect of TRPV1 antagonists on the percentage of embryoid bodies that exhibited spontaneous beating

After the measurement of EB size, these EBs were attached onto gelatin-coated 24-well culture plates to count the percentage of EBs that exhibited spontaneous beating (beating ratio) for 10 consecutive days. The culture media were similar as before but contained either with or without TRPV1 antagonists. Cumulative beating curves from the differentiation day 8 to 17 were constructed (Fig 4C). From the beating curves, it is obvious that the percentage of beating EBs was markedly lower in capsazepine- and SB366791-treated groups than that of control group (Fig 4C). Capsazepine (10 μM) treatment reduced the percentage of beating EBs by >50% from day 8 to day 14. After day 14, the beating ratio gradually recovered, approaching to the control level (Fig 4C). The effect of SB366791 was even more remarkable. Under SB366791 (10 μM) treatment, no beating EBs was observable until the differentiation day 12 (Fig 4C). Starting from day 13, the beating ratio slowly increased but only reached 18% on day 17 (Fig 4C). However, capsaicin (1 μM) treatment had no effect on the percentage of beating EBs.

### Effects of TRPV1 antagonists on the expression of cardiac-specific markers in mESC-CMs

**Fig 5:**
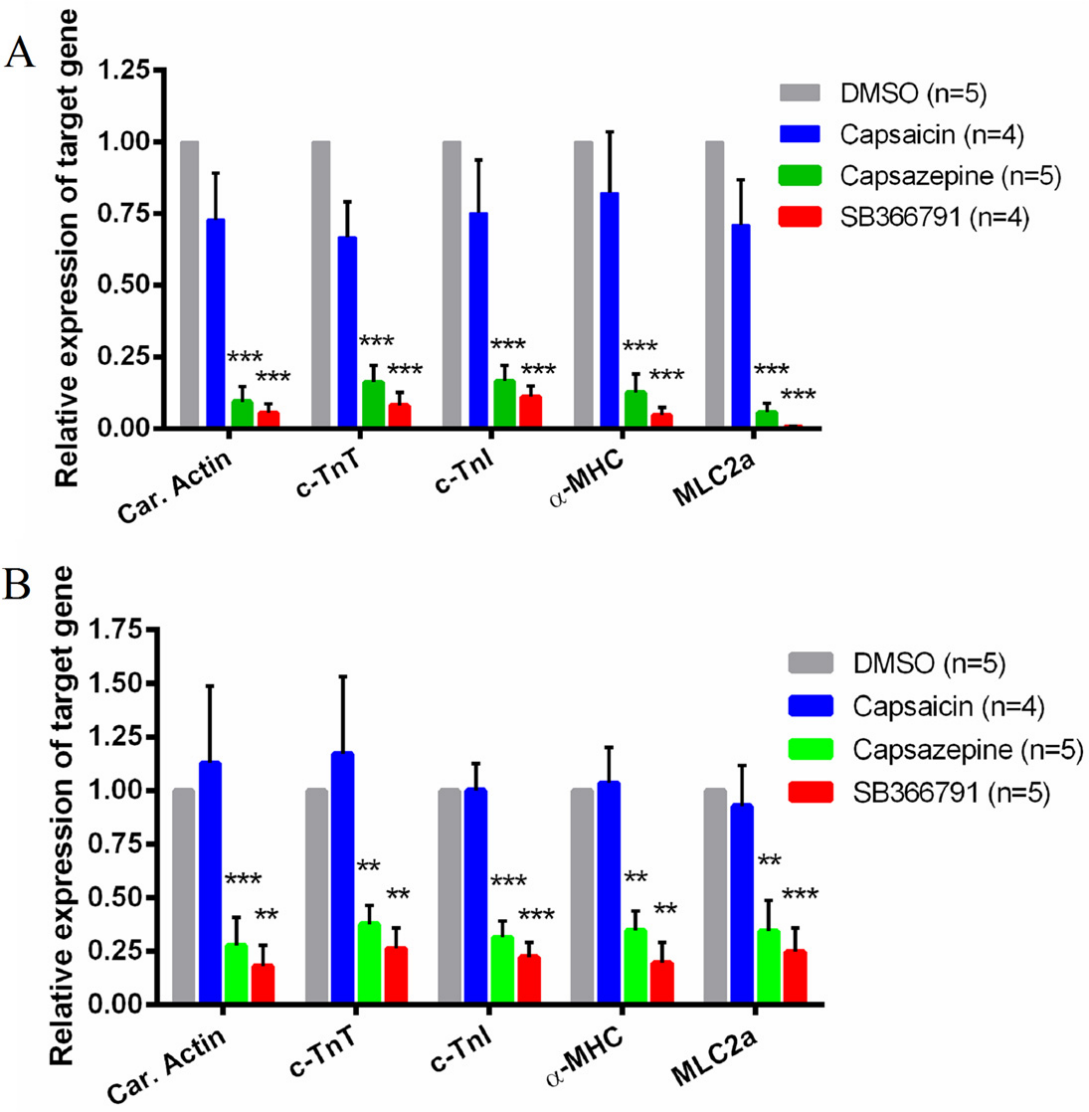
Effect of TRPV1 agonist and antagonists on mRNA expression of cardiac-specific marker genes in mESC-CMs. Shown were the expressional levels of cardiac actin (car. actin), cardiac troponin T (c-TnT), cardiac troponin I (c-TnI) and alpha-myosin heavy chain (α-MHC) after treatment with 0.1% DMSO (vehicle control), 1 μM capsaicin, 10 μM capsazepine, or 10 μM SB366791. (A) qPCR data on the differentiation day 12. (B) qPCR data on the differentiation day 17. Values were Mean ± SEM. n = 4–5 experiments. **P < 0.01, ***P < 0.001 compared to DMSO control.

Quantitative RT-PCRs were used to analyze the expressional level of cardiomyocyte specific marker genes, including cardiac actin, c-TnT, c-TnI and α-MHC [23]. EBs were collected on the differentiation day 12 and 17, and the expression of these marker genes was assessed. Capsazepine (10 μM) treatment reduced the expression of cardiac actin by 91 ± 5% on day 12, and 72 ± 13% on day 17, while SB366791 (10 μM) reduced cardiac actin expression by 94 ± 3% on day 12, and 82 ± 10% on day 17 (Fig 5). Similarly, both capsazepine (10 μM) and SB366791 (10 μM) had strong inhibition on the expression of other three mature cardiomyocyte marker genes c-TnT, c-TnI and α-MHC (Fig 5). However, capsaicin (1 μM) treatment had no significant effect on the expression of all four cardiomyocyte markers either on the differentiation day 12 or day 17 (Fig 5).

### Effects of TRPV1-shRNA on mESC differentiation into cardiomyocytes

**Fig 6:**
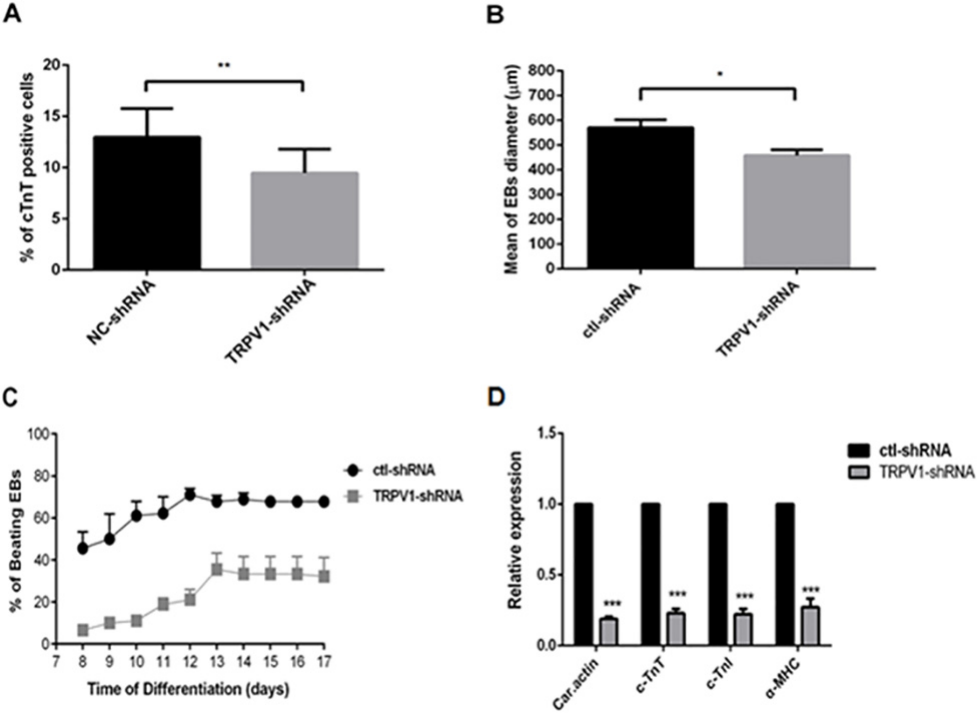
Effect of TRPV1-shRNA on mESC differentiation to cardiomyocytes. (A) Summary of FACS data showing that TRPV1-shRNA reduced the c-TnT-positive cardiomyocytes on the differentiation day 12. (B) Data summary showing the effect of TRPV1-shRNA on EB sizes on the differentiation day 7. Each group contained more than 80 EBs from 3 independent experiments. (C) The effect of TRPV1-shRNA on the percentage of beating EBs. The beating was recorded from differentiation day 8 to day 17. (D) qPCR data on the differentiation day 12, showing the expressional levels of cardiac actin (car. actin), cardiac troponin T (c-TnT), cardiac troponin I (c-TnI) and alpha-myosin heavy chain (α-MHC). ctl-shRNA stands for control scrambled shRNA. Values were Mean ± SEM. n = 3 experiments. *P < 0.05, **P < 0.01, ***P < 0.001.

We further examined the effect of TRPV1-shRNA on mESC differentiation into cardiomyocytes. Results from flow cytometry showed that knockdown of TRPV1 expression with TRPV1-shRNA reduced the percentage of c-TnT-positive cardiomyocytes by ~30% (Fig 6A). Furthermore, TRPV1-shRNA reduced the diameter of EBs (Fig 6B), decreased the percentage of beating EBs (Fig 6C), and suppressed the expression of four cardiomyocyte marker genes (Fig 6D).

## Discussion

The main findings of the present study are as follows: 1) immunostaining showed the expression of TRPV1 proteins in undifferentiated mESCs and mESC-CMs. Quantitative PCRs showed an increase in TRPV1 expression during the differentiation process. 2) TRPV1 agonists capsaicin and camphor elicited a [Ca^2+^]_i_ rise in mESC-CMs, the effect of which was inhibited by TRPV1 antagonist and TRPV1-shRNA. 3) TRPV1 antagonists (capsazepine and SB366791) and TRPV1-shRNA inhibited the growth of EBs and decreased the percentage of beating EBs. 4) TRPV1 antagonists and TRPV1-shRNA also suppressed the expression of cardiomyocyte marker genes, including cardiac actin, c-TnT, c-TnI, and α-MHC. Taken together, these data suggest an important functional role of TRPV1 channels in the differentiation of mESCs into cardiomyocytes.

Capsaicin is a highly-specific TRPV1 agonist, while camphor activates TRPV1, TRPV3 and TRPM8 [24–26]. Among the two TRPV1 antagonists, SB-366791 is highly selective for TRPV1 [27], while capsazepine is a more commonly used [24,28]. In [Ca^2+^]_i_ study, both capsaicin and camphor induced a rise in basal [Ca^2+^]_i_, while in ~50% of mESC-CMs. Because camphor is less specific, we used SB-366791 to further verify the involvement of TRPV1. The results showed that SB-366791 could inhibit the camphor-induced [Ca^2+^]_i_, whil e rise. Knockdown of TRPV1 with TRPV1-shRNA also inhibited the capsaicin- and camphor-induced [Ca^2+^]_i_, whil e rise, further confirming the involvement of TRPV1. These data demonstrated the presence of functional TRPV1 channels in ~50% of mESC-CMs. Note that immunostaining data showed that near all mESC-CMs were TRPV1-positive, while functional data showed that only ~50% of mESC-CMs displayed an increase in [Ca^2+^]_i_ in response to capsaicin or camphor. A likely explanation is that in the rest 50% of mESC-CMs, TRPV1 expression was relatively low so that the responses to capsaicin or camphor could not be detected. Alternatively, TRPV1-mediated Ca^2+^ influx could be offset by other Ca^2+^-lowering events in these cells.

Some TRP isoforms, including TRPV1, TRPC5 and TRPC6, have been reported to regulate cell differentiation in several cell types including neurons, keratinocytes, bone cells and dendritic cells [29–33]. As for TRPV1, its role in cell differentiation appears to be cell type-specific [32,33]. TRPV1 activity was reported to enhance osteoclast and osteoblast differentiation, but inhibit the cytokine-induced differentiation of human dendritic cells [32,33]. However, up to the present, there is no report on the role of TRP channels in cardiomyocyte differentiation. In the present study, we tested the effect of TRPV1 agonists, antagonists and TRPV1-shRNA on three indexes that are associated with cardiomyocyte differentiation, i.e., 1) EB size, 2) the percentage of beating EBs, and 3) the expression of cardiac marker genes. EB size is an index for stem cell proliferation and development; beating EBs represent the presence of contracting cardiomyocytes [34], whereas cardiac actin, c-TnT, c-TnI, and α-MHC are cardiomyocyte markers [23]. Our results showed that TRPV1 antagonists (capsazepine and SB366791) and TRPV1-shRNA reduced the EB size, decreased the percentage of beating EBs, and suppressed the expression of cardiomyocyte-specific marker genes. Together, these data clearly demonstrated that TRPV1 plays a crucial role in the progression of cardiomyocyte differentiation.

Surprisingly, while TRPV1 antagonists and TRPV1-shRNA inhibited the differentiation of mESCs to cardiomyocytes, a TRPV1 agonist capsaicin did not significantly alter the cardiomyocyte differentiation as indicated by its lack of actions on all three differentiation-related indexes. Thus we speculate that TRPV1 may only play a permissive role in mSEC differentiation to cardiomyocytes. It is possible that basal activity of TRPV1 might be already sufficient for this permissive role and that any further increase in TRPV1 activity would not have additional effect.

In conclusion, the present study demonstrated an important role of TRPV1 in the differentiation of mESCs into cardiomyocytes.

## References

[1] Guasch G, Fuchs E. (2005) Mice in the world of stem cell biology. Nat Genet 37: 1201–1206. 16254567 10.1038/ng1667 PMC2405927

[2] Boheler KR, Czyz J, Tweedie D, Yang HT, Anisimov SV, Wobus AM. (2002) Differentiation of pluripotent embryonic stem cells into cardiomyocytes. Circ Res 91: 189–201. 12169644 10.1161/01.res.0000027865.61704.32

[3] Banach K, Halbach MD, Hu P, Hescheler J, Egert U. Caspi O, et al (2003) Development of electrical activity in cardiac myocyte aggregates derived from mouse embryonic stem cells. Am J Physiol Heart Circ Physiol 284: H2114–H2123. 12573993 10.1152/ajpheart.01106.2001

[4] Wobus AM, Guan K, Yang HT, Boheler KR. (2002) Embryonic stem cells as a model to study cardiac, skeletal muscle, and vascular smooth muscle cell differentiation. Methods Mol Biol 185: 127–156. 11768985 10.1385/1-59259-241-4:127

[5] Pucéat M, Jaconi M. (2005) Ca^2+^ signaling in cardiogenesis. Cell Calcium 38: 383–389. 16099501 10.1016/j.ceca.2005.06.016

[6] Yanagida E, Shoji S, Hirayama Y, Yoshikawa F, Otsu K, Uematsu H, et al (2004) Functional expression of Ca^2+^ signaling pathways in mouse embryonic stem cells. Cell Calcium 36: 135–146. 15193861 10.1016/j.ceca.2004.01.022

[7] Kapur N, Mignery GA, Banach K. (2007) Cell cycle-dependent calcium oscillations in mouse embryonic stem cells. Am J Physiol Cell Physiol 292: C1510–C1518. 17092997 10.1152/ajpcell.00181.2006

[8] Wong CK, So WY, Law SK, Leung FP, Yau KL, Yao X, et al (2012) Estrogen controls embryonic stem cell proliferation via store-operated calcium entry and the nuclear factor of activated T-cells (NFAT). J Cell Physiol 227: 2519–2530. 10.1002/jcp.22990 21898397

[9] Wang K, Xue T, Tsang SY, Van Huizen R, Wong CW, Lai KW, et al (2005) Electrophysiological properties of pluripotent human and mouse embryonic stem cells. Stem Cells 23: 1526–1534. 16091557 10.1634/stemcells.2004-0299

[10] Abi-Gerges N, Ji GJ, Lu ZJ, Fischmeister R, Hescheler J, Fleischmann BK. (2000) Functional expression and regulation of the hyperpolarization activated non-selective cation current in embryonic stem cell-derived cardiomyocytes. J Physiol 523: 377–389. 10699082 10.1111/j.1469-7793.2000.t01-2-00377.x PMC2269804

[11] Zhang YM, Shang L, Hartzell C, Narlow M, Cribbs L, Dudley SC Jr. (2003) Characterization and regulation of T-type Ca^2+^ channels in embryonic stem cell-derived cardiomyocytes. Am J Physiol Heart Circ Physiol 285: H2770–H2779. 12919937 10.1152/ajpheart.01114.2002

[12] Yanagi K, Takano M, Narazaki G, Uosaki H, Hoshino T, Ishii T, et al (2007) Hyperpolarization-activated cyclic nucleotide-gated channels and T-type calcium channels confer automaticity of embryonic stem cell-derived cardiomyocytes. Stem Cells 25: 2712–2719. 17656646 10.1634/stemcells.2006-0388

[13] Kleger A, Seufferlein T, Malan D, Tischendorf M, Storch A, Wolheim A, et al (2010) Modulation of calcium-activated potassium channels induces cardiogenesis of pluripotent stem cells and enrichment of pacemaker-like cells. Circulation 122: 1823–1836. 10.1161/CIRCULATIONAHA.110.971721 20956206

[14] Wei WJ, Sun HY, Ting KY, Zhang LH, Lee HC, Li GR, et al (2012) Inhibition of cardiomyocytes differentiation of mouse embryonic stem cells by CD38/cADPR/Ca^2+^ signaling pathway. J Biol Chem 287: 35599–35611. 10.1074/jbc.M112.392530 22908234 PMC3471724

[15] Xia R, Dekermendjian K, Lullau E, Dekker N. (2011) TRPV1: a therapy target that attracts the pharmaceutical interests. Adv Exp Med Biol 704: 637–665. 10.1007/978-94-007-0265-3_34 21290320

[16] Fernandes ES, Fernandes MA, Keeble JE. (2012) The functions of TRPA1 and TRPV1: moving away from sensory nerves. Br J Pharmacol 166: 510–521. 10.1111/j.1476-5381.2012.01851.x 22233379 PMC3417484

[17] Zhang Y, Li L, Hua Y, Nunn JM, Dong F, Yanagisawa M, et al (2012) Cardiac-specific knockout of ET(A) receptor mitigates low ambient temperature-induced cardiac hypertrophy and contractile dysfunction. J Mol Cell Biol 4: 97–107. 10.1093/jmcb/mjs002 22442497 PMC3612005

[18] Zhong B, Wang DH. (2009) Protease-activated receptor 2-mediated protection of myocardial ischemia-reperfusion injury: role of transient receptor potential vanilloid receptors. Am J Physiol Regul Integr Comp Physiol 297: R1681–R1690. 10.1152/ajpregu.90746.2008 19812353 PMC2803628

[19] Tsang SY, Moore JC, Huizen RV, Chan CW, Li RA. (2007) Ectopic expression of systemic RNA interference defective protein in embryonic stem cells. Biochem Biophys Res Commun 357: 480–486. 17434453 10.1016/j.bbrc.2007.03.187 PMC2464293

[20] Law SK, Leung CS, Yau KL, Tse CL, Wong CK, Leung FP, et al (2013) Regulation of multiple transcription factors by reactive oxygen species and effects of pro-inflammatory cytokines released during myocardial infarction on cardiac differentiation of embryonic stem cells. Int J Cardiol 168: 3458–3472. 10.1016/j.ijcard.2013.04.178 23706318

[21] Wobus A, Guan K, Yang HT, Boheler KR. Embryonic stem cells as a model to study cardiac, skeletal muscle, and vascular smooth muscle cell differentiation In: Turksen K, ed. Embryonic Stem Cells: Springer New York, 2002:127–156. 10.1385/1-59259-241-4:127 11768985

[22] Ng SY, Chin CH, Lau YT, Luo J, Wong CK, Bian ZX, et al (2010) Role of voltage-gated potassium channels in the fate determination of embryonic stem cells. J Cell Physiol 224: 165–177. 10.1002/jcp.22113 20333647

[23] Fijnvandraat AC, van Ginneken AC, de Boer PA, Ruijter JM, Christoffels VM, Moorman AF, et al (2003) Cardiomyocytes derived from embryonic stem cells resemble cardiomyocytes of the embryonic heart tube. Cardiovasc Res. 58: 399–409. 12757874 10.1016/s0008-6363(03)00282-7

[24] Xu H, Blair NT, Clapham DE. (2005) Camphor activates and strongly desensitizes the transient receptor potential vanilloid subtype 1 channel in a vanilloid-independent mechanism. J Neurosci 25: 8924–8937. 16192383 10.1523/JNEUROSCI.2574-05.2005 PMC6725586

[25] Selescu T, Ciobanu AC, Dobre C, Reid G, Babes A. (2013) Camphor activates and sensitizes transient receptor potential melastatin 8 (TRPM8) to cooling and icilin. Chem Senses. 38: 563–75; 10.1093/chemse/bjt027 23828908

[26] Moqrich A, Hwang SW, Earley TJ, Petrus MJ, Murray AN, Spencer KS, et al (2005) Andahazy M, Story GM, Patapoutian A Impaired thermosensation in mice lacking TRPV3, a heat and camphor sensor in the skin. Science 307: 1468–1472. 15746429 10.1126/science.1108609

[27] Gunthorpe MJ, Rami HK, Jerman JC, Smart D, Gill CH, Soffin EM, et al (2004) Identification and characterisation of SB-366791, a potent and selective vanilloid receptor (VR1/TRPV1) antagonist. Neuropharmacology 46: 133–149. 14654105 10.1016/s0028-3908(03)00305-8

[28] Phillips E, Reeve A, Bevan S, McIntyre P. (2004) Identification of species-specific determinants of the action of the antagonist capsazepine and the agonist PPAHV on TRPV1. J Biol Chem 279: 17165–17172. 14960593 10.1074/jbc.M313328200

[29] Davis J, Burr AR, Davis GF, Birnbaumer L, Molkentin JD. (2012) A TRPC6-dependent pathway for myofibroblast transdifferentiation and wound healing in vivo. Dev Cell 23: 705–715. 10.1016/j.devcel.2012.08.017 23022034 PMC3505601

[30] Leuner K, Kraus M, Woelfle U, Beschmann H, Harteneck C, Boehncke WH, et al (2011) Reduced TRPC channel expression in psoriatic keratinocytes is associated with impaired differentiation and enhanced proliferation. PLoS One 6: e14716 10.1371/journal.pone.0014716 21364982 PMC3043053

[31] Shin HY, Hong YH, Jang SS, Chae HG, Paek SL, Moon HE, et al (2010) A role of canonical transient receptor potential 5 channel in neuronal differentiation from A2B5 neural progenitor cells). PLoS One 5: e10359 10.1371/journal.pone.0010359 20479868 PMC2866321

[32] Tóth BI, Benko S, Szöllosi AG, Kovács L, Rajnavölgyi E, Bíró T. (2009) Transient receptor potential vanilloid-1 signaling inhibits differentiation and activation of human dendritic cells. FEBS Lett 583: 1619–1624. 10.1016/j.febslet.2009.04.031 19397909

[33] Idris AI, Landao-Bassonga E, Ralston SH. (2010) The TRPV1 ion channel antagonist capsazepine inhibits osteoclast and osteoblast differentiation in vitro and ovariectomy induced bone loss in vivo. Bone 46: 1089–1099. 10.1016/j.bone.2010.01.368 20096813

[34] Mummery CL, Zhang J, Ng ES, Elliott DA, Elefanty AG, Kamp TJ. (2012) Differentiation of human embryonic stem cells and induced pluripotent stem cells to cardiomyocytes: a methods overview. Circ Res 111: 344–358.35. 10.1161/CIRCRESAHA.110.227512 22821908 PMC3578601

